# Kv1.3 Controls Mitochondrial Dynamics during Cell Cycle Progression

**DOI:** 10.3390/cancers13174457

**Published:** 2021-09-04

**Authors:** Jesusa Capera, Mireia Pérez-Verdaguer, María Navarro-Pérez, Antonio Felipe

**Affiliations:** 1 Molecular Physiology Laboratory, Departament de Bioquímica i Biomedicina Molecular, Institut de Biomedicina (IBUB), Universitat de Barcelona, 08028 Barcelona, Spain; jesusa.caperaaragones@kennedy.ox.ac.uk (J.C.); MIP85@pitt.edu (M.P.-V.); navarromarya@gmail.com (M.N.-P.); 2 Kennedy Institute of Rheumatology, University of Oxford, Oxford OX3 7FY, UK; 3 Department of Cell Biology, School of Medicine, University of Pittsburgh, Pittsburgh, PA 15261, USA

**Keywords:** potassium channels, proliferation, adipocytes, mitochondria, fusion/fission

## Abstract

**Simple Summary:**

Voltage-dependent potassium channels control the proliferation of mammalian cells. In addition, mitochondria physiology is highly dynamic during the cell cycle. The aim of this work was to investigate whether the Kv1.3 channel participates in the mitochondrial control of cell cycle progression. Our data confirmed that Kv1.3 facilitates the proliferation of preadipocytes through the control of mitochondrial dynamics. In addition, adipogenesis was also dependent on Kv1.3 expression. We shed light on the role of Kv1.3 in mitochondria and adipose tissue metabolism, contributing further to the control of cell proliferation by Kv1.3.

**Abstract:**

The voltage-gated potassium channel Kv1.3 is a potential therapeutic target for obesity and diabetes. The genetic ablation and pharmacological inhibition of Kv1.3 lead to a lean phenotype in rodents. The mechanism of regulation of body weight and energy homeostasis involves Kv1.3 expression in different organs, including white and brown adipose tissues. Here, we show that Kv1.3 promotes the proliferation of preadipocytes through the control of mitochondrial dynamics. Kv1.3 is expressed in mitochondria exhibiting high affinity for the perinuclear population. The mitochondrial network is highly dynamic during the cell cycle, showing continuous fusion-fission events. The formation of a hyperfused mitochondrial network at the G1/S phase of the cell cycle is dependent on Kv1.3 expression. Our results demonstrate that Kv1.3 promotes preadipocyte proliferation and differentiation by controlling mitochondrial membrane potential and mitochondrial dynamics at the G1 phase of the cell cycle.

## 1. Introduction

The prevalence of the obesity is increasing worldwide. In addition, diabetes, cardiovascular and musculoskeletal diseases, and certain types of cancer are all comorbidities linked to obesity.

Adipose tissue homeostasis is regulated by the combination of adipocyte hyperplasia and hypertrophy. These two phenomena are reciprocally regulated in a sex-, age- and depot-specific fashion [1,2,3]. Changes in adult body weight are due to alterations in adipocyte size rather than cell number. However, adipocyte hyperplasia, which is important during childhood and adolescence, determines the number of adipocytes in adulthood. Thus, obese children have an increased risk of obesity in adulthood [4]. Factors determining preadipocyte proliferation are poorly understood, and most work focuses on adipocyte hypertrophy and adipogenesis. We introduce the voltage-gated potassium (Kv) channel Kv1.3 as an important player in the regulation of preadipocyte proliferation and adipogenesis. The physiological role of Kv1.3 in energy balance is complex but conclusive. Kv1.3−/− mice exhibit a lean phenotype and higher basal metabolic rate [5]. In addition, Kv1.3 gene deletion causes resistance to diet- and genetic-induced obesity [5,6], and pharmacological inhibition of the channel is a promising therapeutic strategy for obesity and diabetes [7,8].

Extensive work has unraveled the mechanisms by which Kv1.3 acts on energy homeostasis. An open debate suggests an intricate scenario, in which Kv1.3 function is orchestrated on different tissues. The olfactory bulb, muscle, immune system and brown (BAT) and white (WAT) adipose tissues are involved. Genetic deletion of Kv1.3 leads to a “super-smeller” phenotype by enhancing olfactory sensitivity and discrimination abilities. The olfactory bulb integrates external (odorants) and internal (i.e., insulin, glucose and leptin) stimuli and projects to different brain regions to regulate energy balance. Interestingly, olfactory bulbectomy prevents the effects of Kv1.3 deletion on body weight, suggesting that the anti-obesity effects of Kv1.3 deletion are dependent on olfactory bulb expression [9]. In addition, pharmacological inhibition of Kv1.3 activates BAT, causing an increase in oxygen consumption, energy expenditure and thermogenesis [8]. Inhibition of Kv1.3 also reduces obesity-induced inflammation in WAT. Furthermore, Kv1.3 is aberrantly expressed in different human pathologies, such as autoimmune diseases and cancer [7,10], and regulates cytokine production in macrophages [11]. Indeed, the inhibition of Kv1.3 significantly reduces TNFα and IL-6 levels in WAT [8,12]. There is also evidence that Kv1.3 has a role in peripheral insulin sensitivity. Kv1.3−/− animals are more sensitive to insulin and exhibit low adiposity. In addition, impairing Kv1.3 function ameliorates the diabetic phenotype [12]. Interestingly, Kv1.3 expression and activity in WAT is insulin-dependent, and it regulates glucose uptake and adipocyte physiology [13]. We reveal a new role for Kv1.3 in the control of body weight through the regulation of preadipocyte proliferation.

The cell cycle-dependent expression and activity of potassium channels trigger signaling cascades, further contributing to the progression of the cell cycle. The subcellular localization of potassium channels varies during cell cycle phases and has important physiological repercussions [14]. In this scenario, Kv1.3 is involved in the cell cycle progression of different cell types [15]. Kv1.3 controls proliferation by means of different ion-dependent and ion-independent mechanisms regulating the G1/S transition [16,17,18,19]. Although the mitogenic role is linked with plasma membrane Kv1.3 localization, recent reports suggest that the mitochondrial channel (mitoKv1.3) is a new modulator of cell cycle progression [20]. MitoKv1.3 regulates apoptosis by controlling the mitochondrial membrane’s potential. Although massive channel inhibition by pro-apoptotic Bax increases reactive oxygen species (ROS levels) and leads to cell death [21], mild mitoKv1.3 pharmacological blockade favors cell cycle progression [20].

Here, we demonstrate that Kv1.3 controls the mitochondrial fusion/fission equilibrium during cell cycle progression by means of a mechanism that likely involves regulation of the mitochondrial membrane potential (∆ᴪ). Kv1.3, which is highly expressed in preadipocyte mitochondria, promotes the formation of a hyperfused mitochondrial network during the G1/S transition. In addition, we found that Kv1.3 is concentrated in perinuclear mitochondria. Our data shed light on new mechanisms of proliferation regulation by intracellular ion channels, thus increasing the repertoire of therapeutic strategies not only for obesity and diabetes but also for cancer and autoimmune diseases.

## 2. Materials and Methods

### 2.1. Cell Culture

3T3-L1 preadipocytes (ATCC) were cultured in DMEM containing 10% NCS (newborn calf serum) at 37 °C in a 7% CO_2_ atmosphere. As indicated, cells were arrested in the G0 phase by serum deprivation in DMEM supplemented with 2% BSA for 24 h. G0-arrested cells were then incubated in the absence or presence of DMEM supplemented with 10% NCS for the indicated times. In some experiments, margatoxin (MgTx, Alomone) or Psora-4 (5-(4-phenylbutoxy) psoralen, Sigma-Aldrich) was added to the media. DMSO (dimethyl sulfoxide) was used as a control and was found to not have any effect. To differentiate adipocytes, postconfluent 3T3-L1 preadipocytes were cultured for 2 days in 10% FBS-supplemented DMEM with 0.25 μM dexamethasone, 5 μg/mL insulin and 0.5 mM IBMX (3-isobutyl-1-methylxanthine). Cells were next transferred to FBS-supplemented DMEM with 5 μg/mL insulin for 2 additional days. Finally, cells were maintained in regular DMEM supplemented with 10% FBS. Fresh medium was added every other day until day 9 to fully differentiated cells.

### 2.2. Lentiviral Infection

To knock down Kv1.3 expression, Kv1.3 shRNA (mouse) lentiviral (LTV) particles were used (Santa Cruz Biotechnology). Infection was performed as previously reported [22] following the manufacturer’s instructions. Briefly, cells (1 × 10^6^) were treated with 50,000 infectious units in the presence of polybrene (2 μg/mL) in 10 cm dishes. Twenty-four hours after transfection, the medium was replaced, and cells were maintained with 10 μg/mL puromycin (Sigma). The specificity of silencing was confirmed by Western blot analysis. Control LTV particles expressing an shRNA with a scrambled sequence were also used.

### 2.3. Cell Cycle Analysis by Flow Cytometry

DNA content was determined with propidium iodide (PI) as reported previously [23]. Cells were collected and spun for 5 min at 200× *g*. Cells washed in 0.5 mL phosphate-buffered saline (PBS) were fixed in ice-cold 70% ethanol for 2 h. Next, cells were washed again, and the pellet was resuspended in 0.5 mL of freshly prepared PI staining solution (20 μg/mL PI, 0.2 mg DNase-free RNase A, 0.1% Triton). Samples were incubated for 30 min at room temperature (RT). Flow cytometric measurements were performed using an Epics XL flow cytometer (Coulter Corporation, Brea, CA, USA), and ploidy analysis was performed using Multicycle software (Gallios, Beckman Coulter).

### 2.4. Proliferation, Viability and Cell Size Assays

Proliferation was analyzed with the Alamar Blue assay [24] as previously described following the manufacturer’s instructions (Life Technologies, Carlsbad, CA, USA). A total of 3000 cells/well were seeded in a 96-well microplate and arrested in the G0 phase by serum deprivation for 24 h. Then, 10% NCS medium containing freshly prepared 10% Alamar blue solution was added. The reduction in resazurin (resorufin) levels in the culture medium was determined at the indicated times by measuring the absorbance at 570 nm (λ_1_) and 600 nm (λ_2_). The results were expressed as the percent difference in reduction with respect to the control cells (wild-type, 0 h) using the following formula:

$$\%\;\mathrm{difference}=\frac{{\left(\varepsilon_{ox}\right)}_{\lambda_{2}}A_{\lambda_{1}}-{\left(\varepsilon_{ox}\right)}_{\lambda_{1}}A_{\lambda_{2}\;}}{{\left(\varepsilon_{ox}\right)}_{\lambda_{2}}A^{{}^{\circ}}{}_{\lambda_{1}}-{\left(\varepsilon_{ox}\right)}_{\lambda_{1}}A^{{}^{\circ}}{}_{\lambda_{2}\;}}\;\cdot 100$$

*A* = absorbance of the test wells
*A*° = absorbance of the control wells

All experiments were performed in triplicate. Cell size and viability were measured by trypan blue exclusion. Briefly, cells were trypsinized and stained with 0.2% trypan blue solution (Life Technologies) and immediately analyzed with a Countess automated cell counter (Invitrogen, Life Technologies).

### 2.5. Protein Extraction and Western Blot Analysis

Cells were washed in cold PBS and lysed on ice with lysis buffer (150 mM NaCl, 1 mM EDTA, 1% Triton X-100, 50 mM Tris–HCl, pH 7.5) supplemented with protease inhibitors (1 μg/mL pepstatin, 1 μg/mL leupeptin, 1 μg/mL aprotinin and 1 mM phenyl-methylsulfonyluoride). Lysates were centrifuged at 16,000× *g* for 15 min at 4 °C, and the protein concentration was measured using the Bradford assay. Protein samples (50 μg) were boiled in Laemmli SDS loading buffer and separated by 10% SDS-PAGE. Next, the samples were transferred to PVDF (polyvinylidene difluoride) membranes (Immobilon-P, Millipore, Burlington, MA, USA), and the membranes were blocked with 5% dry milk supplemented with 0.05% Tween 20 in PBS. The membranes were immunoblotted with the following specific antibodies: anti-Kv1.3 (1/200, Neuromab, Davis, CA, USA), anti-Cyclin E (1/200, Santa Cruz, Dallas, TX, USA), anti-Cyclin D1 (1/200, Santa Cruz, Dallas, TX, USA), anti-Cyclin A (1/200, Santa Cruz, Dallas, TX, USA), anti-β actin (1/50.000, Sigma, Saint Louis, MO, USA), anti-Glut4 (1/500, OSCRX), anti-TIMM50 (1/100, Abcam, Cambridge, UK), anti-Mtf2 (1/1000, Abcam, Cambridge, UK), anti-Drp1 (1/500, Abcam, Cambridge, UK), and anti-Na+/K+ ATPase (1/1000, Dev Studies Hybridoma Bank, University of Iowa, USA). Finally, the membranes were washed with 0.05% Tween 20 PBS and incubated with horseradish peroxidase-conjugated secondary antibodies (BioRad, Hercules, CA, USA).

### 2.6. Purification of Mitochondria

Mitochondria from 3T3-L1 preadipocytes were purified by means of differential centrifugation. Briefly, 80% confluent cells were trypsinized, washed twice with PBS without Ca^2+^ and centrifuged at 600× *g* for 10 min. The cells were homogenized in initial buffer 1 (225 mM mannitol, 75 mM sucrose, 0.1 mM EGTA, 30 mM Tris pH 7.4) and centrifuged again at 600× *g* for 10 min, and the unlysed cells and nuclei were discarded. The supernatant was centrifuged for 10 min at 7000× *g*. The mitochondria-containing pellet was suspended in initial buffer 2 (225 mM mannitol, 75 mM sucrose, 30 mM Tris pH = 7.4) and centrifuged again at 7000× *g*. Pellet isolation was repeated, by means of centrifugation at 10,000× *g*, to obtain the purified mitochondrial fraction. The supernatant obtained after the first round of centrifugation at 7000× *g* was spun further at 20,000× *g* for 30 min, and the pellet was the membranous fraction. The mitochondrial and membranous fractions were suspended in 50 µL of initial buffer 2 and analyzed using WB. All steps were performed at 4 °C.

### 2.7. Immunocytochemistry, Confocal Microscopy and Image Analysis

3T3-L1 cells were seeded on coverslips, washed in PBS without K+ (PBS-K+) and fixed with 4% paraformaldehyde (PFA) for 10 min. The cells were permeabilized using 0.1% Triton X-100 and 20 mM Gly in PBS-K+ for 10 min at RT. After incubation for 60 min in blocking solution (1% BSA, 20 mM Gly, 0.05% Triton X-100, PBS-K+), the cells were treated with a rabbit anti-Kv1.3 antibody (1/20, Alomone) in 1% BSA, 20 mM Gly, and 0.05% Triton X-100 in PBS-K+ and incubated for 90 min. After 3 washes, the preparations were incubated for 60 min with an Alexa Fluor 488-conjugated anti-rabbit antibody (1:200; Molecular Probes), washed and mounted with Mowiol (Calbiochem). All procedures were performed at RT. To visualize mitochondria, the cells were incubated with 500 nM MitoTracker CM-Orange H2TMRos (Thermo Fisher Scientific) in DMEM without serum for 1 h at 37 °C, washed with PBS and fixed with 4% PFA. All images were acquired with a Zeiss 880 confocal microscope.

Colocalization analysis was performed with ImageJ software (National Institutes of Health, Bethesda, MD, USA) as previously described [25]. Mitochondrial perinuclear distribution was automatically analyzed using ImageJ. Briefly, the cell shape and nuclear perimeter were manually defined on preprocessed images. Then, a set of concentric, equally spaced (1.5 µm), enlarged copies of the nuclear perimeter were generated to segment the entire cytoplasmic area into expanding rings. Next, the mitochondrial mean gray values and Pearson coefficients between Kv1.3 and mitochondria were quantified within each ring.

### 2.8. Cell Death Assay and Mitochondrial Membrane Potential Measurement

To evaluate apoptosis, cells were washed with PBS and suspended in FACS buffer (10 mM Hepes, 140 mM NaCl, 2.5 mM CaCl_2_, pH 7.4) containing Annexin V APC and DAPI for 15 min in the dark. The samples were analyzed using a Gallios flow cytometer. To measure the mitochondrial membrane potential by flow cytometry, cells were trypsinized and incubated with 20 nM tetramethylrhodamine methyl ester (TMRM) in DMEM without serum for 30 min at 37 °C. Next, the samples were washed with PBS and analyzed using a Gallios flow cytometer. To measure the mitochondrial membrane potential by confocal imaging, cells were seeded on coverslips, incubated with 500 nM MitoTracker Green for 1 h and 20 nM TMRM for 30 min at 37 °C, washed with PBS and imaged in medium without phenol red at 37 °C in a CO_2_ atmosphere using a Zeiss 880 confocal microscope. The images were analyzed using ImageJ software to obtain the average TMRM intensity per mitochondrial particle for each individual cell. Briefly, MitoTracker Green was used to create a mask to define mitochondrial particles. Next, the TMRM mean intensity was measured for each mitochondrial particle, and the average value per cell was obtained.

### 2.9. Oil Red Staining

3T3-L1 cells were washed twice in PBS, fixed in 4% PFA at RT for 10 min, and then stained with Oil Red O (stock solution: 0.5 g/100 mL dissolved in isopropanol; working solution: 60% Oil Red O stock solution and 40% distilled water) at RT for 1 h. Next, the cells were washed with water, and the stained cellular fat droplets were visualized by light microscopy. For quantitative analysis, the stained lipid droplets were dissolved in 100% isopropanol, and the absorbance at 510 nm was obtained. The values were normalized to the protein content of a duplicate sample. The protein content was quantified with a Pierce BCA assay kit (Thermo Scientific, Waltham, MA, USA) following the manufacturer’s instructions. The absorbance at 562 nm was measured.

### 2.10. Glucose Uptake

3T3-L1 adipocytes were used after 9 days of differentiation. Cells were cultured in Krebs–Ringer–HEPES (KRH) buffer (in mM: 137 NaCl, 4.7 KCl, 2.5 CaCl_2_, 1.18 KH_2_PO_4_, 1.15 MgSO_4_, 20 HEPES, pH 7.4) with or without 10 μM insulin for 30 min at 37 °C. Where indicated, 100 nM margatoxin was added 15 min before the insulin treatment. Glucose uptake started by adding 5 mM 2-deoxyglucose and 0.1 mCi/mL 2-deoxy[H^3^] glucose for 15 min. Next, transport was stopped by adding cold STOP solution containing 50 mM D-glucose in PBS. Cells were lysed for 1 h in 0.1 N NaOH, 0.1% SDS, and radioactivity was measured. Glucose transport was expressed per mg of protein (Bradford assay). The values are expressed relative to the control.

### 2.11. Transmission Electron Microscopy (TEM)

Cells were fixed with 4% PFA and 0.1% glutaraldehyde at room temperature for 1 h followed by 2% PFA for 30 min. High-pressure freeze cryofixation with liquid N2 and cryosubstitution, Lowicryl resin embedding, polymerization of blocks, and cutting of ultrathin sections of 60 nm were performed at the Unitat de criomicroscòpia electrònica (CCiT, University of Barcelona). The samples were mounted on Formvar-coated grids, and the sections were finally contrasted with uranyl acetate 2% for 15 min. Immunolabeling was performed with primary antibodies against Kv1.3 (1:5, Neuromab). The secondary antibodies were conjugated to 18 nm gold particles. The samples were imaged using a Tecnai Spirit 120 kV microscope.

### 2.12. Statistics

The values are the means ± SEs. Significance was analyzed using Student’s *t*-test, one-way ANOVA or two-way ANOVA (GraphPad, PRISM 4.0) as appropriate. A value of *p* < 0.05 was considered significant.

## 3. Results

### 3.1. Kv1.3 Participates in the Proliferation and Differentiation of 3T3-L1 Preadipocytes

**Figure 1:**
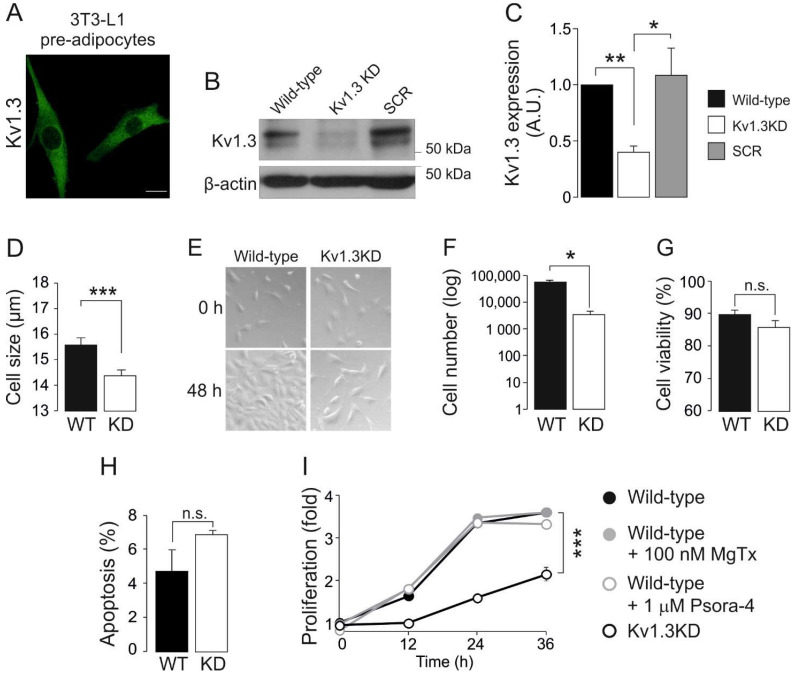
Kv1.3 participates in the proliferation of preadipocytes. 3T3-L1 preadipocytes express Kv1.3, and genetic ablation of the channel alters cell proliferation. (**A**) Representative immunofluorescence confocal image of Kv1.3 in 3T3-L1 preadipocytes. The scale bar represents 20 µm. (**B**) Kv1.3 silencing in 3T3-L1 preadipocytes. Cells were infected with Kv1.3 shRNA (Kv1.3KD) or scramble shRNA (SCR) lentivirus. β-actin was used as a loading control. Noninfected 3T3-L1 cells were called wild-type (WT) cells. (**C**) Quantification of the efficiency of Kv1.3 silencing. The data are the mean ± SE (*n* ≥ 3). * *p* < 0.05, ** *p* < 0.01 (one-way ANOVA and post hoc Tukey test for multiple comparisons). (**D**) Cells were trypsinized, and their size was analyzed with an automatic cell counter. Kv1.3 shRNA (KD). The data are the mean ± SE (*n* = 16). *** *p* < 0.001 (Student’s *t*-test). (**E**) Representative bright field images showing 3T3-L1 preadipocytes. An equal number of cells were seeded and cultured in the absence of growth factors for 24 h. Cells were imaged at 0 and 48 h after the addition of 10% NCS. Note the lower confluence achieved after 48 h by Kv1.3KD preadipocytes than wild-type preadipocytes. (**F**) The cell number in the presence of NCS for 48 h. An equal number of cells were seeded and counted after 48 h in the presence of 10% NCS. The data are the mean ± SE (*n* = 4). * *p* < 0.05 (Student’s *t*-test). (**G**) Cell viability was analyzed with an automatic cell counter by using Trypan Blue staining. The data are the mean ± SE (*n* = 16). (**H**) Percentage of apoptotic cells, as determined by the annexin V assay. No differences were observed between WT and Kv1.3KD preadipocytes (n.s.). The data are the mean ± SE (*n* = 3). (**I**) The proliferation rate of 3T3-L1 preadipocytes, as determined by the Alamar blue assay. An equal number of cells were seeded, and the reduction in the Alamar blue reagent level was measured at 0, 12, 24 and 36 h after serum readdition. Wild-type preadipocytes were treated with or without 100 nM MgTx and 1 μM Psora-4. The data are the mean ± SE (*n* = 4). *** *p* < 0.001 (two-way ANOVA).

Adipocyte differentiation induces Kv1.3 expression and localization in caveolae, which contribute to insulin signaling in mature adipocytes [5,13]. However, the role of this channel in adipose tissue, and by extension in type II diabetes and obesity, is controversial. To further decipher the contribution of Kv1.3 to adipocyte physiology, we generated a Kv1.3-deficient 3T3-L1 preadipocyte cell line. 3T3-L1 preadipocytes were transduced with lentiviral particles containing shRNA against Kv1.3 (Kv1.3KD). Scrambled shRNA was used as a negative control (SCR). Kv1.3 expression was evenly distributed in 3T3-L1 wild-type (WT) preadipocytes (Figure 1A), but showed a 2-fold reduction in Kv1.3KD cells (Figure 1B,C).

Kv channels contribute to controlling cell volume and proliferation. In fact, quiescent cells, characterized by a low proliferative rate and low basal metabolism, exhibit a small cell size [26]. Thus, genetic ablation of Kv1.3 diminished the cell size (Figure 1D) and the proliferation of 3T3-L1 preadipocytes (Figure 1E,F). The reduction in cell number was due to impaired proliferation rather than enhanced cell death because no significant differences in cell viability and apoptosis were observed (Figure 1G,H).

Potassium channels participate in cell cycle progression by means of ion-dependent and ion-independent mechanisms [14]. Therefore, we evaluated the proliferation of preadipocytes in the presence of Kv1.3 inhibitors (Figure 1I), such as 100 nM MgTx and 1 μM Psora-4. Unlike Kv1.3KD, the inhibitors caused no changes in the proliferation of WT and SCR preadipocytes. These observations suggest that Kv1.3 participates in the cell cycle progression of preadipocytes by means of flux-independent mechanisms.

An increase in adipocyte number and hyperplasia results from both the proliferation and differentiation of preadipocytes. Thus, we also investigated the effects of Kv1.3 deficiency on adipogenesis. Kv1.3KD 3T3-L1 preadipocytes reached confluence at a slower rate than WT and SRC cells (Figure 1I). Once confluence was reached, differentiation into adipocytes was induced, and Kv1.3KD 3T3-L1 cells had impaired adipogenesis with fewer and smaller lipid droplets than WT and SCR cells, as shown by Oil Red staining (Figure S1A,B). Because the induction of glucose transporter 4 (Glut4) is a hallmark of adipogenesis [27], the expression of Glut4 and the insulin-induced uptake of glucose were analyzed (Figure S1C,D). Kv1.3KD cells showed reduced Glut4 expression upon differentiation. Furthermore, Kv1.3 was essential for proper insulin-dependent glucose uptake in adipocytes (Figure S1D). As expected, insulin caused a 2.5-fold increase in glucose transport in WT adipocytes, which was partially halted by MgTx [13]. However, Kv1.3KD cells showed no insulin-induced glucose transport.

### 3.2. Kv1.3 Expression Facilitates the G1/S Transition

**Figure 2:**
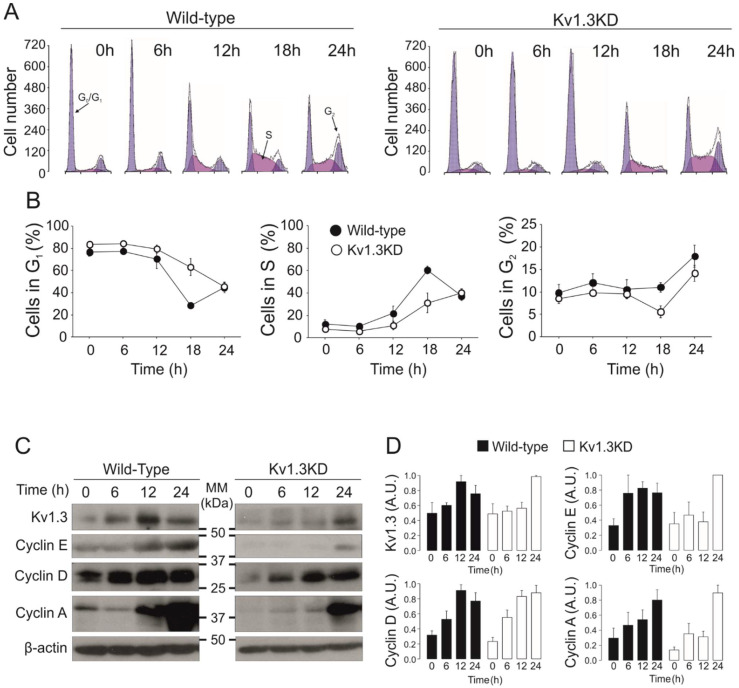
Kv1.3 facilitates the G1/S transition of the cell cycle in preadipocytes. Serum-starved resting cells were incubated for the indicated time after serum readdition. (**A**) Cell cycle analysis of 3T3-L1 preadipocytes was performed with propidium iodide. Representative histograms at 0, 6, 12, 18 or 24 h after serum readdition. The cells exhibit two blue peaks corresponding to the G0/G1 (left) and G2 (right) phases. The cell population in purple corresponds to cells in the S phase. Left panels, wild-type preadipocytes; right panels, Kv1.3KD preadipocytes. (**B**) The % of cells in the G0/G1 phase, % of cells in the S phase and % of cells in the G2 phase for wild-type (black) and Kv1.3KD (white) preadipocytes. The data are the mean ± SE (*n* = 4–10 independent experiments). Two-way ANOVA indicated *p* < 0.001 in the G0/G1 phase and *p* < 0.01 in the S phase between wild-type and Kv1.3KD preadipocytes. (**C**) protein expression of Kv1.3, cyclin E, cyclin D1 and cyclin A. (**D**) Relative protein expression from the data in C. The values were relative to 0 h for each condition and normalized to the level of β-actin, which was used as a loading control (wild-type, black columns; Kv1.3KD, white columns). The data are the mean ± SE (*n* ≥ 3). *p* < 0.05 between WT and Kv1.3KD at 12 h in Kv1.3 and cyclin E (Student’s *t* test).

The activity and expression of many K+ channels is cell cycle-dependent [14]. To further elucidate the effect of Kv1.3 during cell cycle progression, we performed cell cycle analysis on WT and Kv1.3KD preadipocytes (Figure 2). Cells were serum starved, and the number of cells in each phase of the cell cycle at different times was analyzed after serum readdition. Starvation arrested 80% of cells in the G0/G1 phase. Upon serum readdition, WT preadipocytes re-entered the cell cycle, and the percentage of cells in the G0/G1 phase decreased at 18 h with a concomitant increase in the S phase. A further increase in the number of cells in the G2/M phase at 24 h indicated that WT preadipocytes progressed in the cell cycle. In contrast, the percentage of Kv1.3KD preadipocytes in the G0/G1 and S phases indicated a delayed G1/S transition during cell cycle progression. An absence of sub-G0/G1 peaks indicated that the level of apoptosis was negligible (Figure 2A,B).

Next, we analyzed channel expression at different times after serum readdition (Figure 2C,D). Kv1.3 protein abundance transiently peaked at 12 h, corresponding to the G1/S phase of the cell cycle, in WT cells (Figure 2A,B). As expected, Kv1.3 expression was constantly low in Kv1.3KD preadipocytes but rose at 24 h (Figure 2C,D). Cyclins exhibit cell cycle-dependent expression and regulate cell cycle progression. While cyclin D1 and cyclin E peaked in the G1 phase (12 h, G1/S transition), cyclin A expression increased in the G2 phase (24 h) in WT cells upon serum readdition (Figure 2C,D). In contrast, cyclin D1 and E expression steadily increased for 24 h in Kv1.3KD preadipocytes. These data further support a delayed G1/S phase transition, as suggested by cell cycle analysis.

### 3.3. Cell Cycle-Dependent Mitochondrial Kv1.3 Targeting

**Figure 3:**
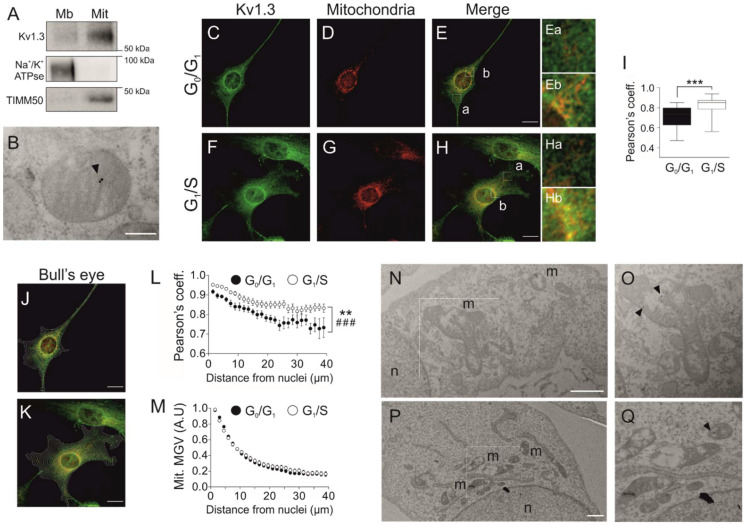
Kv1.3 accumulates at perinuclear mitochondria during the G1/S transition. (**A**) Subcellular fractionation of 3T3-L1 wild-type preadipocytes to obtain the membranous (Mb) and mitochondrial (Mit) fractions. The samples were probed for Kv1.3, Na+/K+ ATPase (a membrane marker) and TIMM50 (a mitochondrial marker). (**B**) Electron micrograph showing mitochondria of 3T3-L1 wild-type preadipocytes. Kv1.3 was labeled with 18 nm immunogold particles (black arrowhead) and was located at the inner mitochondrial membrane. The scale bar represents 200 nm. (**C**–**H**) Cells were either in the G0/G1 or the G1/S phase following serum deprivation or serum readdition for 12 h, respectively. Representative confocal images showing Kv1.3 and mitochondria in wild-type preadipocytes fixed in the G0/G1 (**C**–**E**) and G1/S (**F**–**H**) phase. Ea-Eb and Ha-Hb are magnified images of E and H, respectively. Ea and Ha show distal regions, and Eb and Hb show perinuclear regions. Yellow indicates colocalization of Kv1.3 (green) and mitochondria (red). The scale bar represents 20 µm. (**I**) Pearson’s coefficient of colocalization between Kv1.3 and mitochondria. The data are the mean ± SE (*n* > 30). *** *p* < 0.001. (**J**,**K**) Bull’s eye analysis of the images in panels C–E and F–H. Equally spaced concentric rings of the shape of the nucleus covering the whole cell were automatically drawn. Yellow indicates colocalization between Kv1.3 (green) and mitochondria (red). (**L**) Analysis of Kv1.3 and mitochondrial colocalization. Pearson’s coefficient was measured for each ring and plotted against the distance from the nucleus. The data are the mean ± SE (*n* > 30 cells). ** *p* < 0.01 (G0/G1 vs. G1/S); ###, *p* < 0.001 (vs. distance from the nucleus) (two-way ANOVA). (**M**) Analysis of mitochondrial perinuclear distribution. The mean gray value (MGV) of the mitochondrial marker was measured for each ring and plotted against the distance from the nucleus. The data are the mean ± SE (*n* > 60). (**N**–**Q**) Electron micrographs of cells show Kv1.3 labeled with 18 nm immunogold particles (black arrowheads). The square insets are magnified images of O and Q. n, nuclei. m, mitochondria. The scale bar represents 1 µm.

Although Kv1.3 is essential for the proliferation and differentiation of preadipocytes, plasma membrane Kv1.3 activity does not participate in the control of cell proliferation (Figure 1 and Figure S1). Mitochondrial Kv1.3 controls cell proliferation in cancer cells [20]. Therefore, we wondered whether mitoKv1.3 participated in the control of the cell cycle in 3T3-L1 preadipocytes. Kv1.3 was present in mitochondria (Figure 3A) located at the inner membrane (Figure 3B).

Because the expression of Kv1.3 correlated with the G1/S transition of the cell cycle, we next analyzed mitoKv1.3 levels in cells arrested at the G0/G1 and G1/S transitions after serum readdition. The Pearson coefficient (0.71 ± 0.02) between Kv1.3 and mitochondria was notably high in G0/G1-arrested cells (Figure 3C–E,I). Interestingly, the coefficient rose to 0.82 ± 0.02 (*p* < 0.001) at the G1/S transition, indicating that Kv1.3 expression was augmented in mitochondria during this phase (Figure 3F–H,I). 

Further visual analysis suggested that mitochondrial Kv1.3 expression was not homogeneous. Kv1.3 was concentrated in perinuclear mitochondria (Figure 3E(a,b),H(a,b)). Thus, we performed bull’s eye analysis (Figure 3J–M). Kv1.3 mitochondrial localization decreased with the distance from the nucleus during the G0/G1 and G1/S transitions (Figure 3J–L). In addition, the affinity of Kv1.3 for perinuclear mitochondria was enhanced during the G1/S phase (Figure 3L). However, no differences were found in mitochondrial distribution throughout the cell cycle (Figure 3M). Moreover, electron microscopy confirmed Kv1.3 localization in perinuclear mitochondria (Figure 3N–Q).

### 3.4. Kv1.3 Promotes Hyperfusion of the Mitochondrial Network during the G_1_/S Transition

**Figure 4:**
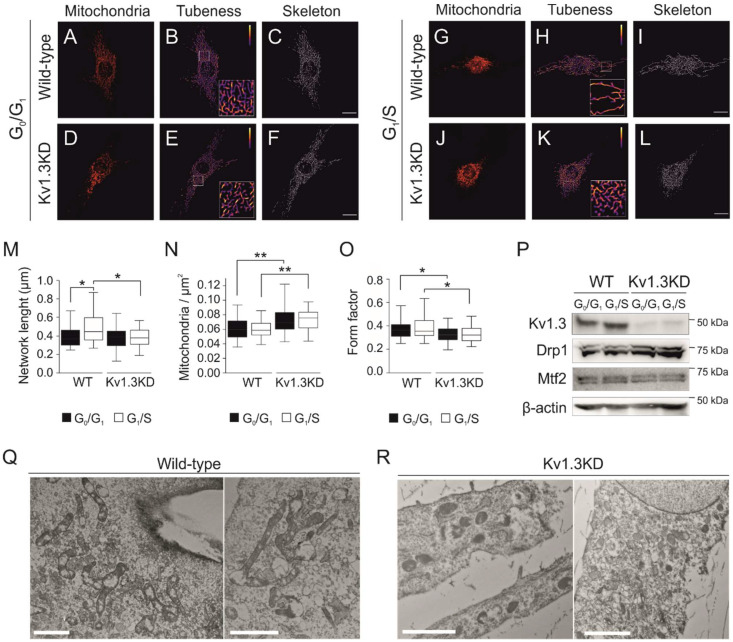
Kv1.3 regulates the mitochondrial fusion/fission equilibrium during the G1/S transition. Confocal images showing mitochondria in cells fixed in the G0/G1 (**A**–**F**) and G1/S (**G**–**L**) phase for WT (**A**–**C**,**G**–**I**) and Kv1.3KD preadipocytes (**D**–**F**,**J**–**L**). The scale bar represents 20 µm. Images were processed (tubeness and skeleton) to perform morphometric analysis of mitochondria. (**M**) The length of mitochondrial networks was measured as the average area of the skeletonized binary image. (**N**) Number of mitochondrial particles per µm^2^. (**O**) The form factor describes the particle shape complexity and was computed as the average (perimeter)2/(4π·area). A circle corresponds to a minimum value of 1. The data are the mean ± SE (*n* > 30). *, *p* < 0.05; **, *p* < 0.01 (one-way ANOVA). (**P**) Protein expression of Kv1.3, dynamin-related protein 1 (Drp1) and mitofusin 2 (Mtf2) during the G0/G1 and G1/S phase in WT and Kv1.3KD 3T3-L1 preadipocytes. β-actin was used as a loading control. (**Q**,**R**) Electron micrographs showing mitochondria of WT (**Q**) and Kv1.3KD (**R**) 3T3-L1 preadipocytes during the G1/S phase. Note that, unlike those in Kv1.3KD, mitochondria in wild-type cells were highly fused.

Cell cycle progression remodels the mitochondrial network, and a transient hyperfused mitochondrial network is needed for a proper G_1_/S transition [28]. Elimination of Kv1.3 impaired mitochondrial dynamics (Figure 4). Thus, mitochondria were hyperfused during the G_1_/S transition in WT preadipocytes (Figure 4A–C,G–I) but not in Kv1.3KD cells (Figure 4D–F,J–L), as quantified by the length of the network (Figure 4M). Concomitantly, the number of mitochondria (mitochondria/μm^2^) was higher and mitochondria were more rounded (form factor closer to 1) in Kv1.3KD cells than in WT cells (Figure 4N–O), with no remodeling during cell cycle progression in Kv1.3KD cells.

Disruption of mitochondrial dynamics in Kv1.3KD preadipocytes during cell cycle progression could be caused by either an increase in fission or the inhibition of mitochondrial fusion at the G_1_/S transition [29]. Thus, we analyzed the expression of mitochondrial shaping proteins, such as mitofusin 2 (Mtf2) and dynamin-related protein 1 (Drp1). Mtf2 participates in mitochondrial fusion, whereas Drp1 promotes fission. Although Mtf2 and Drp1 expression apparently remained stable when WT preadipocytes progressed to the G1/S phase, Drp1 exhibited high expression in Kv1.3KD cells (Figure 4P). TEM confirmed that mitochondria of WT preadipocytes were highly fused at the G_1_/S transition. In contrast, the mitochondria of Kv1.3KD preadipocytes were smaller, and the network was fragmented (Figure 4Q,R).

**Figure 5:**
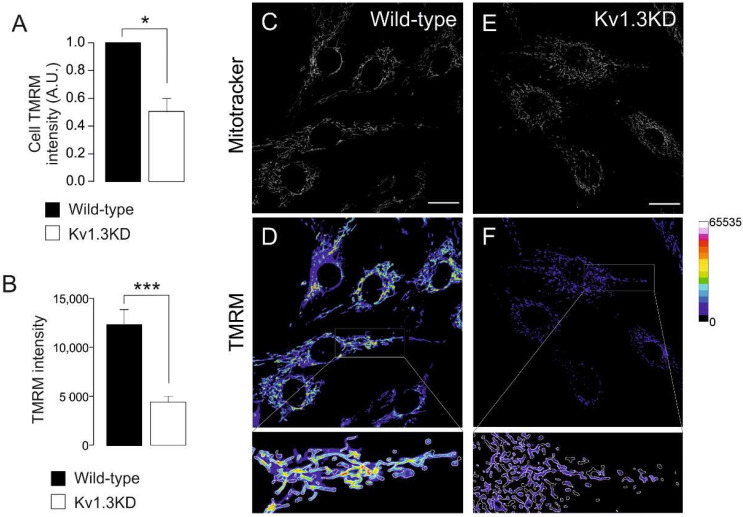
Kv1.3 regulates the mitochondrial membrane potential during the cell cycle. Ablation of Kv1.3 impairs the mitochondrial membrane potential. (**A**) TMRM intensity in wild-type (black bar) and Kv1.3KD (white bar) 3T3-L1 preadipocytes was analyzed with flow cytometry. The data are the mean ± SE (*n* = 3), * *p* < 0.05 (Student’s *t*-test). (**B**) TMRM intensity, as determined by confocal imaging. The data are the mean ± SE (*n* > 30 cells), ***, *p* < 0.001 (Student’s *t*-test). (**C**–**F**) Representative confocal images showing MitoTracker and TMRM intensity in wild-type (**C**,**D**) and Kv1.3KD (**E**,**F**) cells. MitoTracker green (**C**–**E**) was used to create a mask to detect mitochondrial particles, and the mean intensity was analyzed in the TMRM channel (**D**–**F**). The color scale on the right represents TMRM intensity values. The scale bar represents 20 µm. Bottom panels represent 3× magnification of the insets in D and F. The white insets are magnified images of the regions at the bottom.

Kv1.3 deficiency greatly affected mitochondrial dynamics during cell cycle progression. Therefore, we wondered whether the mitochondrial membrane potential was also altered (Figure 5). The mitochondrial membrane potential was analyzed during the G_1_/S transition by TMRM. Mitochondria from WT and Kv1.3KD cells were identified by MitoTracker staining (Figure 5C,E). Mitochondria from Kv1.3KD cells were dramatically depolarized (Figure 5A,B,D,F). These results further suggest that Kv1.3 participates in mitochondrial dynamics in cell cycle progression during proliferation.

## 4. Discussion

Kv1.3 is involved in energy homeostasis in the body and participates in the physiology of different tissues, such as the olfactory bulb [9,30], skeletal muscle, BAT [8] and WAT [13]. Adipocyte Kv1.3 physiology is associated with insulin signaling. We describe a role for Kv1.3 in preadipocyte proliferation, which involves mitochondrial network dynamics. This new and undiscovered mechanism of action links ion channels, mitochondrial physiology and cell proliferation. Kv1.3 accumulates in perinuclear mitochondria, controlling the mitochondrial fusion/fission equilibrium and membrane potential. Thus, it is required for the transient hyperfusion of the mitochondrial network at the G1/S phase transition of the cell cycle. In addition, Kv1.3 is also necessary for proper adipocyte differentiation.

To ascertain the role of Kv1.3 in adipocyte physiology, we generated a Kv1.3 knockdown preadipocyte cell line (Kv1.3KD). Kv1.3KD cells demonstrated dramatically reduced proliferation and adipogenesis. Kv1.3 is involved in the proliferation of many cell types [14,17], and for the first time, we further extended this observation to preadipocytes, linking channel physiology with the onset of obesity. These observations are in accordance with the lean phenotype observed in Kv1.3−/− mice [5] and evidence on isolated adipocytes, in which inhibition of Kv1.3 reduced insulin-dependent glucose uptake [13]. In this context, the ion-dependent and ion-independent roles of Kv1.3 are controversial [16]. White preadipocytes exhibit small potassium currents, which are larger in adipocytes, and high concentrations of tetraethylammonium (TEA) inhibit the proliferation of preadipocytes. However, differentiation is impaired in a dose-dependent manner [31]. This complex scenario suggests that Kv1.3 activity at the plasma membrane of preadipocytes is mainly important for differentiation but not proliferation. Interestingly, Kv1.3 subcellular localization varies during adipogenesis. While Kv1.3 is mainly intracellular in preadipocytes, its expression on the caveolar surface is enhanced during adipogenesis [13]. Thus, our results further suggest a dual role for Kv1.3 that depends on subcellular localization. Intracellular Kv1.3 is important for preadipocyte proliferation, while plasma membrane channels promote adipocyte differentiation. Evidence indicates that Kv1.3 controls the G1/S transition and that channel expression transiently increases at this stage [17,23].

Kv1.3 expression in the inner mitochondrial membrane has been documented [32,33]. However, we provide evidence for heterogeneous mitochondrial Kv1.3 expression. Thus, Kv1.3 was concentrated in perinuclear rather distal mitochondria. It is tempting to speculate that different Kv1.3-containing mitochondrial populations govern different functions. In fact, differential organization of mitochondria has been reported. Subplasmalemmal mitochondria buffer Ca^2+^ movements during T cell activation [34], and ER-contacting mitochondria participate in Ca^2+^ movements and lipid transfer [35]. Evidence indicates that a reciprocal relationship between dysregulated ion transport and mitochondria architecture may contribute to some cancers [36]. On the other hand, mito-nuclear communication has emerged as a new field of research to ascertain different crosstalk mechanisms between these organelles [37]. Clusters of perinuclear mitochondria trigger the accumulation of nuclear ROS, thereby introducing oxidant epigenetic DNA modifications that alter gene expression [38,39]. Other mechanisms controlling gene expression through the mitochondria-nucleus axis include the regulation of Ca^2+^ signaling and ATP levels [37]. Interestingly, Kv1.3 regulates mitochondrial function by controlling the mitochondrial membrane potential, respiration and ROS production [21,33]. Thus, we speculate that cell cycle-dependent accumulation of Kv1.3 in perinuclear mitochondria is involved in the control of mito-nuclear communication. By regulating mitochondrial function, Kv1.3 favors G1/S progression by promoting the expression of genes, such as cyclins. In this context, mitochondria regulate the expression of cell cycle-related genes, such as cyclin E, that promote cell cycle entry [28,40,41].

The cell cycle is a process that requires a large amount of energy, and mitochondria fuel its progression. Interestingly, mitochondria fuse transiently to form a giant tubular network during the G1/S transition, which favors ATP production [42,43]. In this scenario, Kv1.3 is required and evidence would partially suggest non-conducting mechanisms [44]. Thus, the mitochondria of Kv1.3KD cells were smaller and unable to fuse during the G1/S transition. Interestingly, the mitochondria of Kv1.3−/− mice are smaller and unable to adapt and enlarge when challenged by diet-induced obesity [45]. Thus, evidence suggests a key role for Kv1.3 in stress adaptation through modulation of mitochondrial dynamics.

Altered mitochondrial morphology was concomitant with a dramatic loss of mitochondrial membrane potential in Kv1.3KD preadipocytes. Notably, the G1/S transition is highly aerobic and blocked by mitochondrial depolarization. Thus, the fact that Kv1.3KD cells were halted in the G1 phase is consistent with the lower mitochondrial membrane potentials. However, this evidence opens an interesting debate regarding whether mitochondrial depolarization is a consequence of low Kv1.3 activity. Because Kv1.3 mediates an inward K+ current at the inner mitochondrial membrane, a reduction in the mitochondrial membrane’s potential is a consequence and not the cause of the mitochondrial fission generated by the absence of Kv1.3. In fact, mitochondrial function and shape are reciprocally regulated, and the cause-and-effect relationship is under debate. Evidence suggests that fusion requires the proper mitochondrial membrane potential, while fission depolarizes mitochondria [29]. Thus, we hypothesize that Kv1.3 silencing causes remodeling of gene expression by altering mito-nuclear communication. This mechanism promotes mitochondrial fission by enhancing Drp1 expression, which, in turn, leads to a dramatic increase in mitochondrial fission and a reduction in the mitochondrial membrane’s potential.

## 5. Conclusions

**Figure 6:**
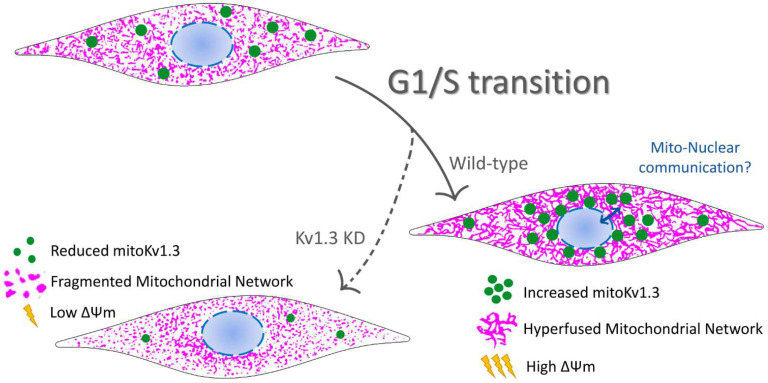
Representative cartoon summarizing the participation of the mitochondrial Kv1.3 (mitoKv1.3) in the proliferation of preadipocytes. Kv1.3 would facilitate the G1/S transition of the cell cycle in preadipocytes accumulating at perinuclear mitochondria. The elucidation of a putative mitochondrial-nuclear communication during this phase of the cell cycle in which Kv1.3 would participate deserves much effort. During the G1/S transition, Kv1.3 would contribute to the mitochondrial fusion/fission equilibrium controlling the mitochondrial membrane potential. Ablation of Kv1.3 (Kv1.3KD) would impair mitochondrial dynamics during cell cycle progression. Kv1.3KD, 3T3-L1 preadipocytes, with a genetic ablation of Kv1.3. Green dots, Kv1.3 channels; magenta, mitochondrial network.

Our results summarized in Figure 6 shed light on the mitochondria-related role of Kv1.3 in controlling cell proliferation. The emerging function of mitochondrial network dynamics in cell cycle progression links mitoKv1.3-dependent events with the control of proliferation exerted by this channel.

## Data Availability

The authors declare that the data generated or analyzed during this study are included in this published article [and its supplementary information files]. In addition, further datasets generated and/or analyzed during the current study are available from the corresponding author on reasonable request.

## Supplementary Materials

The following are available online at https://www.mdpi.com/article/10.3390/cancers13174457/s1, Figure S1: Genetic silencing of Kv1.3 impairs adipogenesis of 3T3-L1 cells.
