# Supplementary material for: Kv1.3 Controls Mitochondrial Dynamics during Cell Cycle Progression
Source: Cancers (Basel). 2021 Sep 4;13(17):4457. doi: 10.3390/cancers13174457 (PMC8431373; doi:10.3390/cancers13174457)
Supplement: Supplementary file 1 [file cancers-13-04457-s001.zip › cancers-1338084-supplementary.pdf]

Supplementary Materials

# Kv1.3 controls mitochondrial dynamics during cell cycle progression

**Jesusa Capera[1,2], Mireia Pérez-Verdaguer[1,3], María Navarro-Pérez[1] and Antonio Felipe[1,*]**

1 Molecular Physiology Laboratory, Dpt. de Bioquímica i Biomedicina Molecular, Institut de Biomedicina (IBUB), Universitat de Barcelona, Spain
2 Kennedy Institute of Rheumatology, University of Oxford, UK.
3 Department of Cell Biology, School of Medicine, University of Pittsburgh, PA, United States.
* Correspondence: afelipe@ub.edu

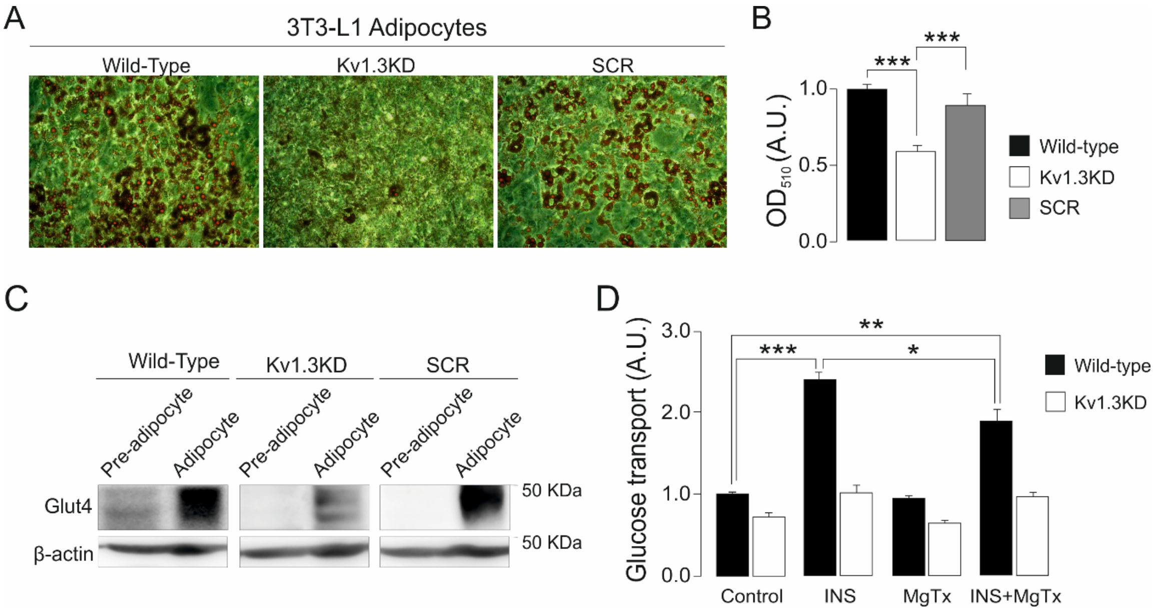


**Figure S1.** *Genetic silencing of Kv1.3 impairs adipogenesis of 3T3-L1 cells.* 3T3-L1 preadipocytes were differentiated into adipocytes. **(A)** Bright field images show Oil Red O staining of differentiated 3T3-L1 adipocytes. **(B)** Quantification of Oil Red O staining of lipids by isopropanol extraction. Optical density (OD) was measured at 510 nm for stained lipids. The data were normalized to total protein amount. The data are the mean ± SE (n=10). ***, $p<0.001$ (one-way ANOVA). **(C)** Protein expression of Glut4 in 3T3-L1 preadipocytes and adipocytes. **(D)** Glucose uptake in wild-type (black columns) and Kv1.3KD (white columns) adipocytes treated with or without insulin (INS) and margatoxin (MgTx). The data are the mean ± SE (n=3). *, $p<0.05$; **, $p<0.01$; ***, $p<0.001$ (one-way ANOVA and post hoc Tukey test for multiple comparisons).
